# S06-3 Supporting our lifelong engagement: mothers and teens exercising (SOLE MATES); from formative research to feasibility testing

**DOI:** 10.1093/eurpub/ckac093.030

**Published:** 2022-08-29

**Authors:** Elaine Murtagh, Méabh Corr, Alyce Barnes, Jaimie McMullen, Philip Morgan

**Affiliations:** 1 Department of Physical Education and Sport Sciences, University of Limerick, Ireland; 2 Kildare and Wicklow Education and Training Board, Wicklow, Ireland; 3 Centre for Active Living and Learning, University of Newcastle, Australia; 4 University of Hawaii

**Keywords:** Adolescent, mother, walking, education, health, female

## Abstract

**Background:**

Health promotion efforts have largely failed to effectively support adolescent girls to meet public health guidelines for physical activity (PA). Engaging mothers in the promotion of PA for their daughters is an important strategy to facilitate behaviour change. This paper provides an overview of the development of the SOLE MATES programme - a novel mother-daughter intervention.

**Methods:**

This programme of research follows the MRC guidance for development and evaluating complex interventions (Craig, 2008). Two reviews were conducted to synthesise the evidence base on (1) mother-daughter PA interventions and (2) adolescent girls' perceptions of PA participation. The behaviour change wheel (BCW) framework was then used to design the components of an intervention to improve adolescent girls' PA (Michie et al, 2011). Finally, a single-arm feasibility trial was conducted to examine recruitment, data collection, acceptability, resources and participant responses.

**Results:**

Our evidence reviews showed that only a limited number of interventions designed exclusively to target mothers and daughters have been conducted. In addition, programmes for adolescent girls should focus on alternative activities aside from the competitive team-based sports often offered.

The BCW design process resulted in a group-based face-to-face intervention, involving six intervention functions (education, persuasion, incentivisation, training, modelling, enablement) and 18 behaviour change techniques. The 6-week programme includes weekly educational and practical group sessions plus home tasks and the provision of participant resources. In addition to encouraging participants to walk regularly, the programme also aims to empower mothers to be role models for their daughters positive health behaviour.

Participants in the feasibility study were mothers (n = 27) with daughters (n = 31) aged 12-16 years. Eligibility rates were 93.4%, and baseline activity levels were low. Programme content, measures and facilitators were acceptable. Daily steps increased in mothers (+2,875, p = 0.009) and daughters (+1,393, p = 0.007).

**Conclusions:**

We demonstrate the value of formative research in the intervention development process. We show that it is feasible to increase the PA levels of teenage girls through an inter-generational multi-component PA programme. The SOLE MATES programme should now be examined for effectiveness using a RCT. If successful the scale-up and sustainability of the programme should be explored.

